# Clinical Reports, with Cases

**Published:** 1882-03

**Authors:** Edward Rushmore

**Affiliations:** Plainfield, N. J.


### CLINICAL REPORTS, WITH CASES.

Edward Rushmore, M. D., Plainfield, N. J.

Certain well-known physicians of experience having lately publicly expressed a disapproval of, or want of interest in the publication of clinical cases, it has occurred to me to state what seems to be sufficient grounds for their publication. The study of the materia medica being at best a notably laborious work, whatever facilitates it should be cherished as a means to the end for which only the profession of medicine can logically and rightly exist, viz.: the healing of the sick. Probably all physicians will admit that their most ready and available knowledge of our remedies is that which has been impressed upon them in the successful application of those remedies; but the necessary limitations to individual experience prevents one from acquiring in this way all the knowledge he may need; and it is superfluous to remark upon the readiness of interchange by clinical reports. Said the venerable Hering: " Let every practitioner report his cases or at least his cured symptoms, and at the great harvest time they will help to separate the true from the false, and a new, much abridged materia medica may be issued, not based upon the arbitrary notions of one, but on the united experience of all." If reports are perverted from their purpose of showing the probably verified symptoms, and lead to the use of a remedy for the *name* of a disease, the fault lies wholly with the perverter and is assuredly no new fault with him. I would therefore ask our master prescribers to remember how much the less experienced stand in need of the knowledge which forms their daily thought. It may be said that all knowledge is in our books, but meeting the same knowledge again in connection with cures gives it more than the excellent force of a common repetition; and it seems further probable that the number of reliable, but as yet purely clinical indications would by the faithful report of cases be increased.

To say that the value of clinical reports depends largely on the fullness with which the concomitants and conditions of the morbid phenomena are stated, is but to repeat a truism in Homœopathy, for there are often the distinctive, and hence decisive elements of a case.

A lack of this fulness in some recent reports makes it impossible to say why some other remedy was not equally well indicated.

We derive additional assurance, if any were needed, of the superiority of prescribing upon the facts of diseases, rather than upon conjectures as to morbid anatomy, founded thereon, in the want of agreement between the pathological writers and in the unceasing succession of their hypotheses.

A report of a few cases is herewith subjoined:

*Drosera* 900 (F.) one dose quickly cured in a fleshy lady, pain in ankle on bending foot, especially in going up and down stairs.

*Aphis chenopodii glauci* CM (F.) cured at once a scraping, tickling and fullness in the throat, causing hawking, in a fleshy lady, soon followed by a heavy, dull griping in abdomen, going to sides, with drawing in occiput—antidoted by *Nux vomica.*

*Capsicum* 900 (F.) cured quickly a case described by letter, as bad catarrh; worse at time of grass-cutting; tickling in nose; eyes swelled with inflammation; weakness.

*Nux vomica* 200 (Dunham) given for four days, cured an ulcer of five or six weeks' standing, on the left lower gum, in an elderly, spare lady; the ulcer sensitive and worse when dyspeptic or fatigued. The patient had dull headache and vertigo early in the morning, going off after breakfast; tasteless, empty eructations after eating, and a feeling of a load at the pit of the stomach.

*Chelidonium* 900 (F.), one dose cured the following: Letters run tegether while reading; left eyelids agglutinated in morning; lachrymation in wind. Allen gives lachrymation in open air. The concomitants were: Pain, from reading, in the eyes; worse from candlelight; also lachrymation from reading. These also were removed, though not in the *materia medica.*

*Ammon. carb.* CM (F.), seven powders, one every morning, cured a long-standing pain in the abdomen below navel, every morning before breakfast; ameliorated by the pressure of the hand, and still more by lying down; accompanied with left inguinal hernia, which also protruded less, and was less sore after taking the medicine.

*Nat. mur.* 900 (F.), followed with *Lycopod.* CM. Swan, cured a long-standing affection in a middle-aged man, as follows: Pain in stomach and abdomen on rising in the morning, dull and heavy or sharp, relieved by empty eructations. Bad-tasting, dark-yellowish, thick mucus in the mouth in the morning. Pain in right shoulder-blade, worse from motion, better from lying on the affected side. Headache occasionally in the morning on rising, lasting all day. Pain in the lower back; worse from walking and stooping. All these symptoms were removed by the remedies mentioned.

*Silicea*, in different high potencies given on two occasions to a lady for dental fistula, relieved the same very much, but was followed both times by a feeling of cold in the head, so that she came to ask me if I had given her anything to give her a cold.

*Pulsatilla* CM (F.), one dose cured immediately and permanently, sleepiessness till after midnight, in a little girl of eight years, who had always slept well till two weeks before she got the medicine. The sleeplessness was attended with weeping, because she could not sleep.

